# *Trichoderma* Species Attract *Coptotermes formosanus* and Antagonize Termite Pathogen *Metarhizium anisopliae*

**DOI:** 10.3389/fmicb.2020.00653

**Published:** 2020-04-09

**Authors:** Chao Wen, Hongpeng Xiong, Junbao Wen, Xiujun Wen, Cai Wang

**Affiliations:** ^1^Guangdong Key Laboratory for Innovation Development and Utilization of Forest Plant Germplasm, College of Forestry and Landscape Architecture, South China Agricultural University, Guangzhou, China; ^2^Integrative Microbiology Research Centre, South China Agricultural University, Guangzhou, China; ^3^Beijing Key Laboratory for Forest Pest Control, College of Forestry, Beijing Forestry University, Beijing, China

**Keywords:** *Trichoderma*, *Coptotermes formosanus*, attractiveness, entomopathogen, antagonism

## Abstract

Although subterranean termites live within soil, little attention has been paid on the potential interaction among subterranean termites and soil microbes. Herein, we conducted different choice tests to investigate aggregation and tunneling behaviors of *Coptotermes formosanus* Shiraki responding to soil/sand treated with conidia of seven soil fungi, *Trichoderma longibrachiatum* Rifai, *Trichoderma koningii* Oud., *Trichoderma hamatum* (Bon.) Bain., *Trichoderma atroviride* Karsten, *Trichoderma spirale* Indira and Kamala, *Trichoderma harzianum* Rifai, and *Trichoderma viride* Pers. ex Fries. In aggregation-choice test, soil treated with nearly all *Trichoderma* species tested (except *T. koningii*) significantly increased termite aggregation compared with untreated soil. In tunneling-choice tests, termites produced significantly larger tunnels in sand treated with *T. longibrachiatum* or *T. koningii* than that in untreated sand. We hypothesized that *Trichoderma* species could benefit termites by protecting them from infection of the entomopathogenic fungus *Metarhizium anisopliae* (Metschn) Sorokin, and three *Trichoderma* species that attracted termites (*T. longibrachiatum*, *T. atroviride*, and *T. harzianum*) were tested. The antagonism tests showed that the three *Trichoderma* species suppressed growth of *M. anisopliae.* Also, the median lethal time (LT_50_) of termites exposed to both *Trichoderma* species and *M. anisopliae* was significantly longer than termites exposed to *M. anisopliae* alone. Interestingly, though significantly fewer termites aggregated in soil treated with *M. anisopliae* conidia compared with untreated soil, *M. anisopliae* conidia mixed with *T. longibrachiatum* or *T. harzianum* were no longer repellent to termites. Our results showed that the fungi in the genus *Trichoderma* (1) exerted generally attractive effects on termites, (2) protected termites from the infection of entomopathogenic fungus, and (3) altered pathogen-avoiding behaviors of termites. Future studies will be required to understand the mechanisms underlying these newly discovered effects.

## Introduction

Subterranean termites usually live in moist habitats that favor the growth of diverse microbes (Cornelius et al., 2012). The interaction among termites and some environmental fungi has been extensively investigated over the past few decades. For example, it is well known that the foraging activities of subterranean termites can be enhanced by the wood-rotting fungi and blue-staining fungi living in their food sources (Amburgey, 1979; Ohmura et al., 1995; Cornelius et al., 2002, 2004; Little et al., 2012a, b, 2013; Clay et al., 2017). Also, termites have evolved multiple strategies to escape entomopathogenic fungi and reduce epidemics within their colonies (Myles, 2002; Shimizu, 2003; Yanagawa et al., 2005; Yanagawa and Shimizu, 2007; Peterson and Scharf, 2016; Davis et al., 2018). Surprisingly, though subterranean termites excavate large amounts of soil and construct extensive tunneling systems underground, the potential interactions between subterranean termites and soil fungi have received little attention.

*Trichoderma* spp. are typical soil fungi that decompose dead wood, litters, or organic matters (Harman et al., 2004; Govarthanan et al., 2018). A few studies reported the commensalism between *Trichoderma* species and subterranean termites. For example, Zoberi and Grace (1990) isolated *Trichoderma koningii* Oud., *Trichoderma harzianum* Rifai, and *Trichoderma viride* Pers. ex Fries from the wood infested with *Reticulitermes flavipes* (Kollar). Jayasimha and Henderson (2007) isolated *T. harzianum*, *Trichoderma virens* (Miller, Giddens, and Foster) Arx, *Trichoderma asperellum* Samuels, Lieckf. and Nirenberg, and *Trichoderma ghanense* Doi, Abe, and Sugiyama from the integuments and guts of *Coptotermes formosanus* (Shiraki). Both studies identified *Trichoderma* species using conventional culture-based methods, and more *Trichoderma* species (especially for those that grow slowly or cannot grow well on the artificial media) might be detected in future studies using non-culture-based methods.

Interestingly, some recent studies showed that the interactions between subterranean termites and *Trichoderma* species are more complex than previously thought. For example, Xiong et al. (2018) reported that *C. formosanus* preferred to make tunnels in the soil previously treated with the commercial conidial formulations of *T. harzianum* (BioWorks^®^) or *T. viride* (Shuiguxin^®^) compared with the untreated soil. However, in that study only two commercially available *Trichoderma* species were tested, and it is unclear whether fungi in the genus *Trichoderma* generally attract *C. formosanus*. In this study, we conducted different choice tests to investigate the aggregation and tunneling preferences of *C. formosanus* responding to the soil treated with the unformulated conidia of seven *Trichoderma* species (*Trichoderma longibrachiatum* Rifai, *T. koningii* Oud., *Trichoderma hamatum* (Bon.) Bain., *Trichoderma atroviride* Karsten, *Trichoderma spirale* Indira and Kamala, *T. harzianum* Rifai, and *T. viride* Pers. ex Fries) and untreated soil. Among them, *T. koningii*, *T. harzianum*, and *T. viride* were previously isolated from live termites and their habitats (Zoberi and Grace, 1990; Jayasimha and Henderson, 2007). The remaining *Trichoderma* species are common in soils, and therefore may also affect behaviors of subterranean termites.

Since our studies showed a strong attractive effect of *Trichoderma* species to *C. formosanus* (see section “Results”), it would be valuable to study the biological significance of *Trichoderma* species on termites. Interestingly, *Trichoderma* species are well-known as plant symbionts that can inhibit the growth of many phytopathogenic fungi including *Verticillium dahliae* (Kleb), *Pratylenchus brachyurus* (Godfrey), and *Fusarium graminearum* (Schwabe) (Carrero-Carrón et al., 2016; Kath et al., 2017; Saravanakumar et al., 2018). *Metarhizium anisopliae* (Metschn) Sorokin is a common entomopathogenic fungus that lives in soils and attacks subterranean termites (e.g., Kramm et al., 1982; Zoberi, 1995; Jones et al., 1996; Wang and Powell, 2004; Wright et al., 2005; Denier and Bulmer, 2015). We hypothesized that *Trichoderma* species could suppress entomopathogenic fungi such as *M. anisopliae* and therefore benefit termites. In the present study, the antagonism tests were conducted to investigate whether *Trichoderma* species inhibit the growth of *M. anisopliae* under *in vitro* conditions. We also conducted the mortality tests to study whether *Trichoderma* species protect termites from the infection of *M. anisopliae* under *in vivo* conditions.

Many previous studies showed that entomopathogenic fungi such as *M. anisopliae* repelled termites (Hussain et al., 2010). All of these studies only tested the effect of *M. anisopliae*, but there are many types of soil microbes coexisting within soil under natural conditions. We hypothesize that the coexistence of *Trichoderma* species may disturb the pathogen-avoidance behaviors of termites triggered by *M. anisopliae.* Here, we conducted choice and non-choice tests to compare the aggregation and tunneling behaviors of termites reacting to the soil containing conidia of *M. anisopliae* alone, or containing conidia of both *M. anisopliae* and *Trichoderma* species.

## Materials and Methods

### Termites

Four *C. formosanus* colonies were collected from campus and arboretum of South China Agricultural University (SCAU), Guangzhou, China, using the methods described by Xiong et al. (2018). The collection sites of the four termite colonies were > 500 m from each other. Termites collected from the same colony were transported to the moisture-proof storage container (55 × 40 × 31 cm [L by W by H]) with wet wood sticks, and maintained at room temperature (24 ± 2°C) under darkness for <1 month.

### *Trichoderma* Species and *Metarhizium anisopliae*

Seven *Trichoderma* species (*T. longibrachiatum*, *T. koningii*, *T. harzianum*, *T. hamatum*, *T. atroviride*, *T. viride*, and *T. spirale*) and one entomopathogenic fungus (*M. anisopliae*) were used in this study. *T. longibrachiatum*, *T. atroviride*, *T. hamatum*, *T. spirale*, and *M. anisopliae* were purchased from BIOBW Biotechnology Co., Ltd. (Beijing, China), and *T. harzianum*, *T. koningii*, and *T. viride* were purchased from Guangdong Culture Collection Center (GCCC) (Table 1). Based on the information provided by BIOBW and GCCC, these fungi were identified to the species level using the molecular methods. In brief, a specific fragment of the internal transcribed spacer (ITS) region was amplified using the primers ITS1 (5′-TCCGTAGGTGAACCTGCGG-3′) and ITS4 (5′-TCCTCCGCTTATTGATATGC-3′) (White et al., 1990), and the amplified DNA was sequenced and aligned against sequences of the type strain from the databank (NCBI^1^). All fungi were cultured using potato dextrose agar (PDA) medium in an incubator at 25 ± 1°C. To obtain large amounts of conidia, sterile distilled water (7 mL) was added to the PDA cultures, and the conidial suspension was transferred to a 250 mL Erlenmeyer flask containing autoclaved rice (50 g rice mixed with 50 mL distilled water, and sterilized at 121°C for 20 min). The Erlenmeyer flask was maintained in an incubator at 25 ± 1°C for 5–10 days until the rice medium was covered by large amounts of conidia. Sterile distilled water (200 mL) was added to the Erlenmeyer flask and shook for 3 min using a vortex mixer. The concentration of conidial suspension was determined using a hemocytometer (Shanghai Qijing Biochemical Reagent Instrument Co., Ltd, China). Sterile distilled water was added and mixed with suspensions to obtain required concentrations of conidia to set the bioassays.

**TABLE 1:** Information on fungi used in the present study.

Fungal species	Strain No.	Source	Source of isolation^d^
*Trichoderma longibrachiatum*	Bio-68049^a^	BIOBW^b^	Soybean (*Glycine max* L.)
*Trichoderma koningii*	GIM-3.518	GCCC^c^	Humus soil
*Trichoderma harzianum*	GIM-3.442	GCCC	Humus soil
*Trichoderma hamatum*	Bio-08848	BIOBW	Broad bean (*Vicia faba* L.) soil
*Trichoderma atroviride*	Bio-08876	BIOBW	*Panax notoginseng* (Burkill) rhizosphere
*Trichoderma viride*	GIM-3.432	GCCC	Humus soil
*Trichoderma spirale*	Bio-088439	BIOBW	Humus soil
*Metarhizium anisopliae*	Bio-67986	BIOBW	Soil

*^*a*^*Trichoderma longibrachiatum* strain Bio-68049 is also referred to as *Trichoderma longibrachiatum* strain NBRC 31918, with the ITS sequence available at http://www.nbrc.nite.go.jp/NBRC2/NBRCDispSearchServlet. ^*b*^BIOBW = BIOBW Biotechnology Co., Ltd. (Beijing, China). ^*c*^GCCC = Guangdong Culture Collection Center. ^*d*^Information was obtained from BIOBW and GCCC.*

### Soil/Sand Preparation

Topsoil was collected from the two locations of arboretum of SCAU where *C. formosanus* activities have been detected. Samples of soil were sent to the Laboratory of Forestry and Soil Ecology (College of Forestry and Landscape Architecture, SCAU), and identified as sandy clay loam (70% sand, 9% silt, and 21% clay) and loamy sand soil (78% sand, 14% silt, and 8% clay). Fine sand (Suqian Weiyou Trading Co., Ltd., China) was purchased. Soil and sand were sterilized at 80°C for 3 days, and completely dried at 50°C for >2 weeks. To remove coarse particles, soil was sifted through a 2-mm sieve, and sand was sifted through a 0.85-mm sieve. Required amount of conidial suspensions and sterile distilled water were added to prepare the wet soil (24% (w/w) moisture, calculated using the formula as follows: [(wet weight - dry weight)/dry weight] × 100%) or sand (15% moisture) that contained each of the seven fungi (*T. longibrachiatum*, *T. koningii*, *T. harzianum*, *T. hamatum*, *T. atroviride*, *T. viride*, or *T. spirale*) with certain concentrations of conidia as mentioned in each experiments. To prepare the untreated soil/sand, only sterile distilled water was added.

### Do *Trichoderma* Species Attract *Coptotermes formosanus*?

#### Aggregation-Choice Test

This study aimed to investigate whether *Trichoderma* conidia in soil trigger the aggregation preference by termites. Protocols provided by Xiong et al. (2018, 2019) were modified to prepare the bioassay arenas. In brief, blocks (40 × 40 × 10 mm [L by W by H]) of soil (sandy clay loam) were made using a plastic mold. A filter paper (diameter = 125 mm) was placed on the bottom of a Petri dish (diameter = 140 mm, height = 13.5 mm) and moistened with 2 mL sterile distilled water. A soil block treated with *Trichoderma* conidia (2.5 × 10^7^ conidia/g soil) was placed on one side of the Petri dish, while an untreated soil block was placed on the other side. A piece of balsa wood (20 × 20 × 1 mm [L by W by H]), a common food source of termites used in the laboratory studies, was placed at the center of each soil block. Fifty termites (45 workers and five soldiers) were released at the center of each Petri dish. The bioassays were maintained in an environmental chamber (25 ± 1°C under total darkness). After 24 h, the percentage of termites in each location (either aggregated in/on each soil block or stationing on the filter paper) was recorded. In total, there were seven aggregation-choice tests, and each test was repeated 24 times (six replicates for each termite colony).

#### Tunneling-Choice Test

This study aimed to investigate whether *Trichoderma* conidia in sand trigger the tunneling preference by termites. Methods provided by Xiong et al. (2018, 2019) were modified to prepare the experiments. The bioassay arenas consisted of two square acrylic plates (156 × 156 × 3 mm [L by W by H]), which were assembled with four narrow edge strips (156/150 × 3 × 1.5 mm [L by W by H]) to create a two-dimensional tunneling chamber. A hole (diameter = 5 mm) was made on the central point of the upper plate and the bottom of an acrylic container (diameter = 5 mm, height = 15 mm). The upper plate and acrylic container were attached (the holes were connected) and fixed with hot glue. The tunneling arena was divided equally into two parts. One half was filled with 28 g sand treated with *Trichoderma* conidia (2.5 × 10^7^ conidia/g sand), and the other half was filled with the same weight of untreated sand. Four pieces of balsa wood (10 × 10 × 1 mm [L by W by H]) were placed in the corners of the tunneling arena as the food source. The two plates were then held together with binding clips. Fifty termites (45 workers and five soldiers) were released into the acrylic container. The arenas were placed in an incubator at 25 ± 1°C under total darkness. After 2 days, the arenas were horizontally placed on a LED panel light. A small square of graph paper (12 × 12 mm, 10 lines per centimeter) was placed on the bottom plate as the scale, and a high-resolution picture was taken. The area of tunnels made by termites in the *Trichoderma*-treated and untreated sand was measured using the Image J software (US National Institutes of Health, Bethesda, MD, United States). In total, there were seven tunneling choice tests, and each test was repeated 24 times (six replicates for each termite colony).

### Do *Trichoderma* Species Suppress Termite Pathogen?

#### Antagonism Test

We investigated whether *Trichoderma* species (*T. longibrachiatum*, *T. harzianum*, or *T. atroviride*) inhibit the growth of *M. anisopliae* using both dual-culture and fermentation filtrate experiments. In the dual culture experiments, the fungal discs (diameter = 4 mm) were obtained by punching the edge of each fungal colony (after 4 days of initial inoculation) using a sterile hole puncher. A fungal disc of *M. anisopliae* was placed at one side of the PDA medium (10 mm distance from the edge of the Petri dish), while a fungal disc of each of the three *Trichoderma* species was placed on the other side. The PDA media with the *M. anisopliae* disc alone was used as the controls. The radial diameter of *M. anisopliae* colonies was measured using a digital caliper until its diameter no longer changed (measurement data unchanged for 3 days). Inhibitory rate was determined using the formula as follows: inhibitory rate (%) = [(mean radial diameter of *M. anisopliae* in the controls – radial diameter of *M. anisopliae* in each antagonism test)/mean radial diameter of *M. anisopliae* in the controls] × 100%. Each test was repeated five times.

In the fermentation filtrate tests, the activated mycelial plugs of each *Trichoderma* species were inoculated to 300 mL potato dextrose broth (PDB, Guangdong Huankai Microbial Technology Co., Ltd., Guangdong, China) in Erlenmeyer flasks, and were then incubated on a shaker (120 r/min, 25 ± 1°C) for 10 days. The contents in flask were centrifuged for 5 min at 47,000 *g*, and the supernatant was filtered through a 0.22 μm membrane filter. The filtered supernatant (1 mL) or the same amount of sterile distilled water (control) was then mixed with PDA medium (19 mL). Before solidification, the mixtures (20 mL) were poured into the Petri dishes (diameter = 90 mm). After the medium was solidified, a fungal disc (diameter = 4 mm) of *M. anisopliae* was placed at the center of each medium. The diameter of mycelial growth of *M. anisopliae* was measured until its diameter no longer changed. Inhibitory rate was calculated using the same formula as mentioned in the dual-culture experiments. Each test was repeated five times.

#### Mortality Test

This study aimed to investigate whether *Trichoderma* species protect termites from the infection of *M. anisopliae.* Three *Trichoderma* species (*T. longibrachiatum*, *T. harzianum*, and *T. atroviride*) that attracted termites (see section “Results”) were tested in this and the following experiments. A sterilized filter paper (diameter = 85 mm) was placed on the bottom of the Petri dish (diameter = 90 mm). There were eight treatments: treatment 1–4: only conidial suspension of each *Trichoderma* species (*T. longibrachiatum*, *T. harzianum*, or *T. atroviride*) or *M. anisopliae* (1 mL conidial suspension at the concentration of 5 × 10^7^ conidia/mL) was evenly added onto the filter paper (the final concentration of each fungus was 5 × 10^7^ conidia/dish); treatment 5–7: both conidial suspensions of each *Trichoderma* species (*T. longibrachiatum*, *T. harzianum*, or *T. atroviride*) and *M. anisopliae* (0.5 mL conidial suspension for each fungus at the concentration of 1 × 10^8^ conidia/mL) were evenly added onto the filter paper (the final concentration of each fungus was 5 × 10^7^ conidia/dish); and treatment 8: only sterile distilled water (1 mL) was evenly added onto the filter paper (controls).

Fifty termites (45 workers and five soldiers) were released into each Petri dish. The bioassays were maintained in an environmental chamber at 25 ± 1°C under total darkness for 20 days. The mortality of termites (termites were considered dead when their body laid sideways or upside-down) in each replicate was recorded each day. Each treatment was repeated 24 times (six replicates for each termite colony).

### Do *Trichoderma* Species Alter the Repellency of *Metarhizium anisopliae* Against *Coptotermes formosanus*?

#### Aggregation-Choice Test

This study aimed to investigate whether *Trichoderma* species alters the repellency of *M. anisopliae* against *C. formosanus*. Similar procedures of the aggregation-choice tests described earlier were used to prepare soil blocks (loamy sand soil) and set the bioassays, but the treated soil blocks contained conidia of *Trichoderma* species or *M. anisopliae* alone or together (Table 2) at a final concentration for each fungus of 1 × 10^7^ conidia/g soil. The percentages of termites in each location (aggregated in/on each soil block or stationing on the filter paper) were calculated at 12 h. Each choice test was repeated 12 times (three replicates for each termite colony).

**TABLE 2:** Aggregation-choice tests to investigate whether *Trichoderma* species alters the repellency of *Metarhizium anisopliae* against *Coptotermes formosanus.*

Test	Control soil block	vs.	Treated soil block
1	Untreated soil	vs.	Soil only containing conidia of *T. longibrachiatum*
2	Untreated soil	vs.	Soil only containing conidia of *T. harzianum*
3	Untreated soil	vs.	Soil only containing conidia of *T. atroviride*
4	Untreated soil	vs.	Soil only containing conidia of *M. anisopliae*
5	Untreated soil	vs.	Soil containing both conidia of *M. anisopliae* and *T. longibrachiatum*
6	Untreated soil	vs.	Soil containing both conidia of *M. anisopliae* and *T. harzianum*
7	Untreated soil	vs.	Soil containing both conidia of *M. anisoplia*e and *T. atroviride*

*The final concentration for each fungus was 1 × 10^7^ conidia/g soil.*

#### Tunneling Non-Choice Test

This study aimed to investigate the tunneling behaviors of termites in response to sand treated with *M. anisopliae* alone, or *M. anisopliae* and *Trichoderma* species together. The bioassay arenas were two-dimensional tunneling chambers as described earlier, but the arena was not divided into two parts. Instead, each tunneling arena was filled with 56 g sand which (1) only contained each of the four fungi (*T. longibrachiatum*, *T. harzianum*, *T. atroviride*, or *M. anisopliae*) with the final concentration of 1 × 10^7^ conidia/g sand; (2) contained both conidia of *Trichoderma* species (*T. longibrachiatum*, *T. harzianum*, or *T. atroviride*) and *M. anisopliae* at the final concentration of 1 × 10^7^ conidia/g sand for each fungus; (3) did not contain any fungal conidia (untreated sand). Fifty termites (45 workers and five soldiers) were released to the acrylic container. After 7 days, the areas of tunnels were measured as described earlier. In addition, the number of termites alive in each arena was recorded, and the excavation volume of sand was estimated by weighing the sand that was transported into the release chamber. Each treatment was repeated 12 times (three replicates for each termite colony).

### Statistical Analyses

For the aggregation-choice tests, the log-ratio transformation was conducted to make the compositional data (percentages) independent (Kucera and Malmgren, 1998; Wang et al., 2015; Xiong et al., 2018). The transformed data were compared using two-way analysis of variance (ANOVA) with termite colony as the random effect and location as the fixed effect. For the tunneling tests, the areas of tunnels in the treated and untreated sand in the choice tests, and the mortality, weight of excavated sand, and tunnel area in the non-choice tests were analyzed using two-way ANOVA with colony group as the random effect and the treatment as the fixed effect. For the antagonism tests, the inhibitory rates of the three *Trichoderma* species against *M. anisopliae* were compared using one-way ANOVA. For the mortality tests, the mortality of termites was compared using two-way ANOVA with termite colony as the random effect and treatment as the fixed effect. In addition, the median lethal time (LT_50_) of termites in each treatment was calculated and compared using the probit analysis (IBM SPSS Statistics version 22.0, Chicago, IL, United States). Tukey’s HSD tests were conducted for multiple comparisons after each ANOVA at α = 0.05.

## Results

### Do *Trichoderma* Species Attract *Coptotermes formosanus*?

#### Aggregation-Choice Tests

The mean survival of termites was >95% in the aggregation-choice test. Significantly more termites preferred to aggregate in/on the soil block containing conidia of *T. longibrachiatum*, *T. harzianum*, *T. hamatum*, *T. atroviride*, *T. viride*, or *T. spirale* compared with the untreated soil blocks (Table 3). However, there was no significant difference in percentage of termites between soil blocks treated with *T. koningii* and untreated ones (Table 3).

**TABLE 3:** Percentage (mean ± SE) of termites aggregated in the *Trichoderma-*treated or untreated soil blocks or stationing on the Petri dishes.

Test	Treated block	Petri dish	Untreated block	Statistical result			Effect
				*F*	d.f.	*P*	
*T. longibrachiatum*	67.33 ± 5.06 a	9.88 ± 1.34 b	22.79 ± 5.05 b	38.35	2, 60	<0.0001	Attractive
*T. koningii*	44.88 ± 5.97 a	21.33 ± 2.47 b	33.79 ± 5.08 ab	4.00	2, 60	0.0235	N.A.
*T. harzianum*	65.25 ± 5.58 a	12.17 ± 2.22 b	22.58 ± 4.84 b	38.66	2, 60	<0.0001	Attractive
*T. hamatum*	70.00 ± 4.45 a	11.00 ± 1.71 b	19.00 ± 4.09 b	62.33	2, 60	<0.0001	Attractive
*T. atroviride*	64.08 ± 5.12 a	19.50 ± 3.56 b	16.42 ± 3.84 b	59.12	2, 60	<0.0001	Attractive
*T. viride*	51.17 ± 5.45 a	22.42 ± 2.48 b	26.42 ± 4.86 b	7.20	2, 60	0.0016	Attractive
*T. spirale*	81.42 ± 3.12 a	8.00 ± 1.52 b	10.58 ± 2.46 b	135.64	2, 60	<0.0001	Attractive

*Different letters within the same row indicate significant differences (*P* < 0.05). Attractive effects indicate that significantly more termites aggregated in/on the soil blocks treated with conidia of each *Trichoderma* species compared with untreated soil. N.A. indicates no significant difference in percentages of termites when compared between *Trichoderma*-treated and untreated soil blocks.*

#### Tunneling-Choice Test

Termites produced significantly larger tunnels in sand treated with *T. longibrachiatum* or *T. koningii* than that in untreated sand. However, the areas of tunnels were similar in untreated sand and sand treated with *T. harzianum*, *T. hamatum*, *T. atroviride*, *T. viride*, or *T. spirale* (Table 4).

**TABLE 4:** Areas of tunnels (mean ± SE) produced in *Trichoderma*-treated and untreated sand.

Test	Treated sand (mm^2^)	Untreated sand (mm^2^)	Statistical result			Effect
			*F*	d.f.	*P*	
*T. longibrachiatum*	1129.96 ± 72.28a	706.22 ± 58.08b	23.93	1, 40	<0.0001	Enhanced
*T. koningii*	1163.89 ± 47.27a	908.31 ± 89.60b	11.56	1, 40	0.0015	Enhanced
*T. harzianum*	1030.26 ± 77.14a	1043.34 ± 60.13a	0.02	1, 40	0.8816	N.A.
*T. hamatum*	881.38 ± 62.82a	862.43 ± 57.18a	0.06	1, 40	0.8011	N.A.
*T. atroviride*	1027.45 ± 84.75a	937.84 ± 54.04a	0.97	1, 40	0.3313	N.A.
*T. viride*	929.92 ± 74.52a	837.54 ± 60.25a	1.00	1, 40	0.3232	N.A.
*T. spirale*	811.14 ± 55.60a	959.28 ± 71.32a	2.64	1, 40	0.1121	N.A.

*Different letters within the same row indicate significant differences (*P* < 0.05). “Enhanced” indicates that significantly larger tunnels were produced by termites in sand treated with conidia of each *Trichoderma* species compared with untreated sand. “N.A.” indicates no significant difference in tunnel areas when compared between *Trichoderma*-treated and untreated sand.*

### Do *Trichoderma* Species Suppress Termite Pathogen?

#### Antagonism Test

**FIGURE 1:**
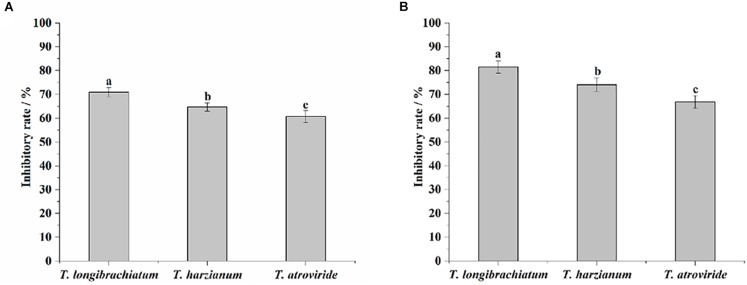
Inhibitory rates of the three *Trichoderma* species against *Metarhizium anisopliae* in the dual-culture tests **(A)** and fermentation filtrate experiments **(B)**. Different letters indicate significantly different (*P* < 0.05).

In the dual-culture tests, the average inhibitory rates of *T. longibrachiatum*, *T. harzianum*, and *T. atroviride* were 71.0, 64.8, and 60.7%, respectively. *T. longibrachiatum* exhibited a significantly stronger inhibitory effect against *M. anisopliae*, as compared with *T. harzianum* and *T. atroviride* (*F* = 29.48, df = 2, *P* < 0.0001; Figure 1A). In the fermentation filtrate tests, the average inhibitory rates of *T. longibrachiatum*, *T. harzianum*, and *T. atroviride* were 81.5, 74.0, and 66.9%, respectively. The inhibitory rate of *T. longibrachiatum* against *M. anisopliae* was significantly higher than that of *T. harzianum*, and both were significantly higher than that of *T. atroviride* (*F* = 37.41, df = 2, *P* < 0.0001; Figure 1B).

#### Mortality Test

**FIGURE 2:**
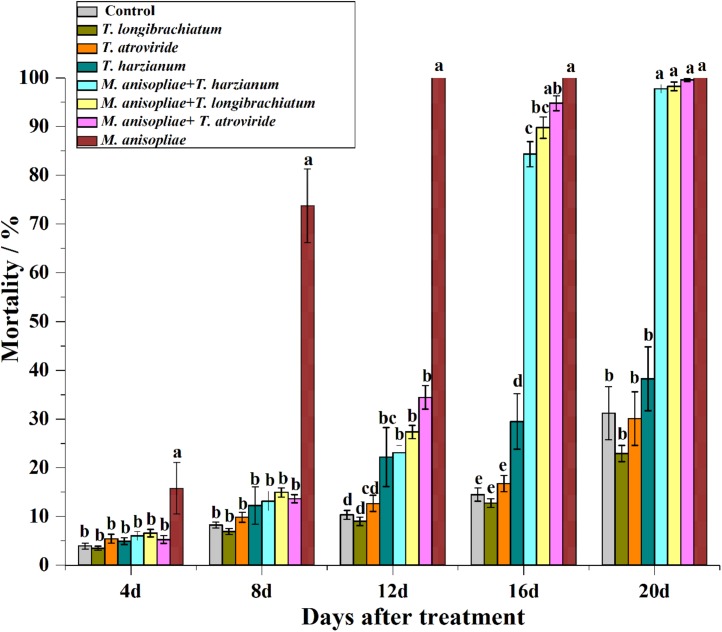
Mortality (means ± SE) of termites at 4, 8, 12, 16, and 20 days after they were introduced into the Petri dishes containing the filter paper treated with sterilize distilled water (control), or conidial suspension of *Trichoderma longibrachiatum*, *Trichoderma atroviride*, *Trichoderma harzianum*, *Metarhizium anisopliae*, or both *Metarhizium anisopliae* and each of the three *Trichoderma* species. Different letters indicate significantly different within each day (*P* < 0.05).

From day 4 to 14, the mortality of termites exposed to *M. anisopliae* alone was significantly higher than in the other treatments (Figure 2; statistical results are shown in Supplementary Table S1). From day 17 to 20, however, the mortality of termites exposed to *M. anisopliae* alone was not significantly different from termites that were exposed to both *Trichoderma* species and *M. anisopliae* (Figure 2 and Supplementary Table S1), but all of them had significantly higher mortality compared with termites exposed to *Trichoderma* conidia alone or distilled water (controls). In addition, for the four termite colonies, the LT_50_ value of termites exposed to *M. anisopliae* alone was significantly lower than termites exposed to both *M. anisopliae* and *Trichoderma* species (Table 5).

**TABLE 5:** Median lethal time (LT_50_) of termites exposed to conidia of *Metarhizium anisopliae* alone, or both conidia of *Metarhizium anisopliae* and each *Trichoderma* species.

Colony	Treatment	*n*	LT_50_ (days)	Regression equation	χ^2^ (df)
1	*M. anisopliae* alone	300	5.55 (5.25–5.84)	*y* = −4.93 + 0.89x	673.34 (118)
	*M. anisopliae* and *T. longibrachiatum*	300	11.84 (11.52–12.17)	*y* = −5.56 + 0.47*x*	425.02 (118)
	*M. anisopliae* and *T. harzianum*	300	12.83 (12.50–13.18)	*y* = −5.17 + 0.40*x*	393.13 (118)
	*M. anisopliae* and *T. atroviride*	300	11.88 (11.51–12.24)	*y* = −6.04 + 0.51*x*	596.45 (118)
2	*M. anisopliae* alone	300	5.57 (5.14–5.98)	*y* = −4.44 + 0.80*x*	1197.59 (118)
	*M. anisopliae* and *T. longibrachiatum*	300	12.74 (12.39–13.09)	*y* = −5.10 + 0.40*x*	418.70 (118)
	*M. anisopliae* and *T. harzianum*	300	12.70 (12.31–13.09)	*y* = −6.50 + 0.51*x*	677.41 (118)
	*M. anisopliae* and *T. atroviride*	300	11.11 (10.79–11.42)	*y* = −7.01 + 0.63*x*	565.57 (118)
3	*M. anisopliae* alone	300	6.28 (5.79–6.75)	*y* = −4.48 + 0.71*x*	1394.21 (118)
	*M. anisopliae* and *T. longibrachiatum*	300	12.32 (11.98–12.67)	*y* = −5.22 + 0.42*x*	427.70 (118)
	*M. anisopliae* and *T. harzianum*	300	12.60 (12.20–13.00)	*y* = −4.91 + 0.39*x*	520.14 (118)
	*M. anisopliae* and *T. atroviride*	300	11.70 (11.33–12.07)	*y* = −5.36 + 0.46*x*	544.88 (118)
4	*M. anisopliae* alone	300	7.21 (6.93–7.49)	*y* = −6.51 + 0.90*x*	621.04 (118)
	*M. anisopliae* and *T. longibrachiatum*	300	13.30 (12.96–13.66)	*y* = −5.10 + 0.38*x*	393.44 (118)
	*M. anisopliae* and *T. harzianum*	300	13.75 (13.37–14.15)	*y* = −5.26 + 0.38*x*	461.06 (118)
	*M. anisopliae* and *T. atroviride*	300	11.44 (11.10–11.78)	*y* = −6.19 + 0.54*x*	543.82 (118)

### Do *Trichoderma* Species Alter the Repellency of *Metarhizium anisopliae* Against *Coptotermes formosanus*?

#### Aggregation-Choice Test

The mean survival of termites was >94% in each aggregation-choice test. Significantly fewer termites were found in/on the soil blocks treated with the conidia of *M. anisopliae* compared with untreated ones (Table 6), indicating a repellent effect of *M. anisopliae* against termites. However, percentage of termites aggregated in/on the soil treated with both *M. anisopliae* and *T. longibrachiatum* or *T. harzianum* was not significantly different from the untreated ones (Table 6).

**TABLE 6:** Percentage (mean ± SE) of termites aggregated in/on the treated (containing conidia of *Trichoderma* species or *Metarhizium anisopliae* alone or together) or untreated soil blocks, or stationing on the Petri dishes.

Test	Treated block	Petri dish	Untreated block	Statistical result			Effect
				***F***	**d.f.**	***P***	
*T. longibrachiatum* alone	64.14 ± 4.16a	9.66 ± 1.24c	26.20 ± 4.19b	43.10	2, 10	<0.0001	Attractive
*T. harzianum* alone	62.20 ± 5.22a	22.89 ± 4.11b	14.91 ± 2.39b	22.00	2, 10	<0.0001	Attractive
*T. atroviride* alone	51.61 ± 6.23a	12.77 ± 3.97b	35.62 ± 4.86ab	22.76	2, 10	<0.0001	N.A.
*M. anisopliae* and *T. longibrachiatum*	42.62 ± 6.84a	8.74 ± 0.90b	48.64 ± 6.89a	20.32	2, 10	<0.0001	N.A.
*M. anisopliae* and *T. harzianum*	25.87 ± 7.04a	7.95 ± 1.56b	66.18 ± 7.29a	47.78	2, 10	<0.0001	N.A.
*M. anisopliae* and *T. atroviride*	22.30 ± 4.01b	12.14 ± 1.85c	65.56 ± 4.10a	56.80	2, 10	<0.0001	Repellent
*M. anisopliae* alone	15.00 ± 3.20b	15.09 ± 2.89b	65.95 ± 4.97a	25.72	2, 10	<0.0001	Repellent

*Different letters within the same row indicate significant differences (*P* < 0.05). Attractive effects indicate that significantly more termites aggregated in/on the treated soil blocks compared with untreated ones. Repellent effects indicate that significantly fewer termites aggregated in/on the treated soil blocks compared with untreated ones. N.A. indicates no significant difference in percentages of termites when compared between treated and untreated soil blocks.*

#### Tunneling Non-Choice Test

**FIGURE 3:**
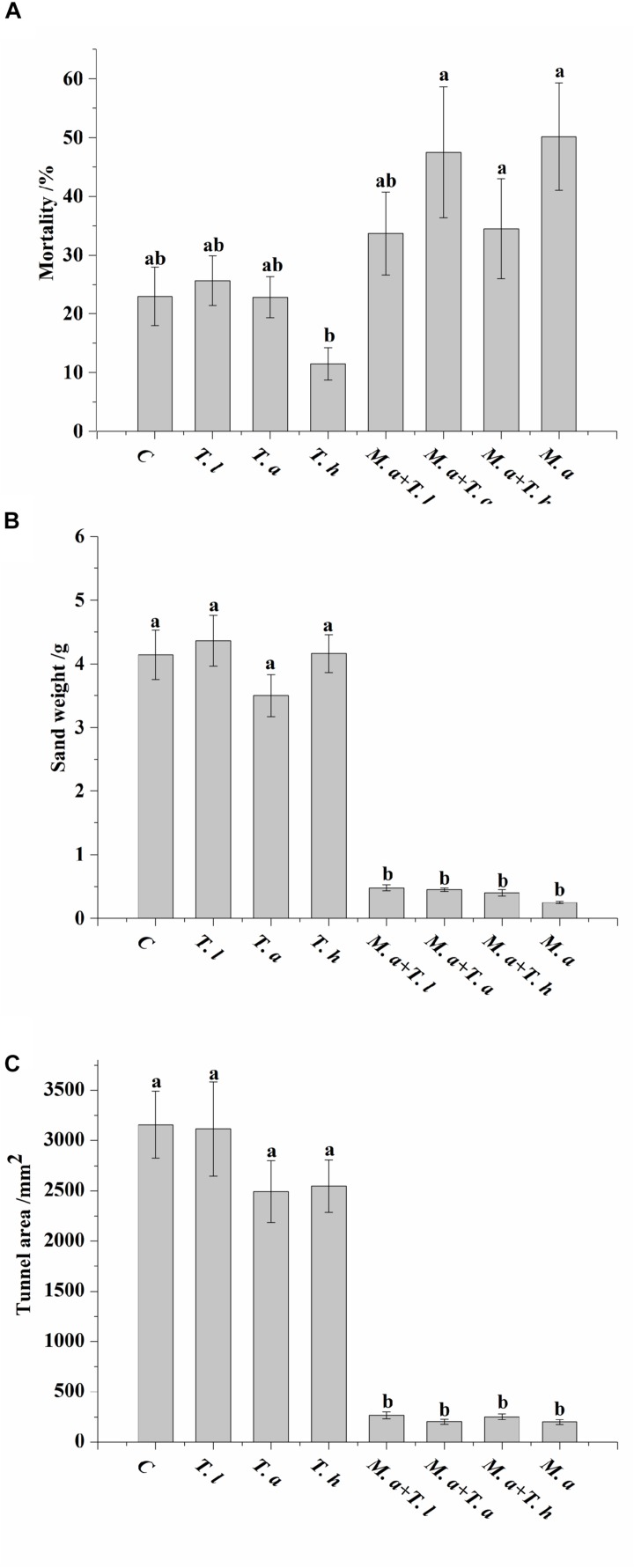
Mortality of termite **(A)**, weight of sand transported into the release chamber **(B)**, and area of tunnels produced by termites **(C)** are shown as mean ± SE. Different letters indicate significantly differences (*P* < 0.05). C = control sand (sand treated with sterile distilled water); *T. l* = sand treated with conidia of *Trichoderma longibrachiatum*; *T*. *h* = sand treated with conidia of *Trichoderma harzianum*; *T. a* = sand treated with conidia of *Trichoderma atroviride*; *M. a* + *T. l* = sand treated with both conidia of *Metarhizium anisopliae* and *Trichoderma longibrachiatum*; *M. a* + *T. h* = sand treated with both conidia of *Metarhizium anisopliae* and *Trichoderma harzianum*; *M. a* + *T. a* = sand treated with both conidia of *Metarhizium anisopliae* and *Trichoderma atroviride*; *M. a* = sand treated with conidia of *Metarhizium anisopliae.*

Termites had significantly higher mortality when exposed to sand treated with conidia of *M. anisopliae* (either alone or together with *T. harzianum* or *T. atroviride* species) compared with the sand treated with conidia of *T. harzianum* alone (*F* = 5.94, df = 7, 64, *P* < 0.0001; Figure 3A). In addition, significantly less weight of excavated sand (*F* = 66.62, df = 7, 64, *P* < 0.0001; Figure 3B) and area of tunnels (*F* = 39.80, df = 7, 64, *P* < 0.0001; Figure 3C) were found when the sand was treated with conidia of *M. anisopliae* (either alone or together with *Trichoderma* species) compared with the untreated sand or sand treated with each *Trichoderma* species alone.

## Discussion

**FIGURE 4:**
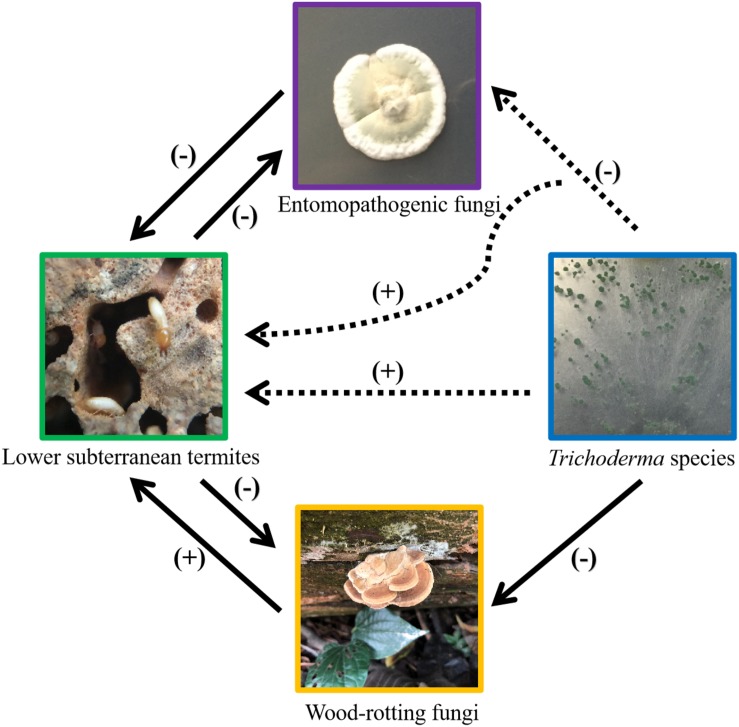
Current understanding of the complex interactions among lower subterranean termites, wood-rotting fungi, termite pathogen, and *Trichoderma* species. The solid-line arrows indicate published results, and the dashed-line arrows indicate results shown in the present study. “(+)” represents positive effects (i.e., attract termites or provide antimicrobial protection to termites) and “(−)” represents negative effects (i.e., kill or repel termites, or suppress the growth of fungus). Pictures were taken by the first author.

Our study showed that *Trichoderma* species in substrates (soil or sand) generally attracted *C. formosanus.* In addition, some *Trichoderma* species benefited termites by inhibiting *M. anisopliae*. Interestingly, *T. longibrachiatum* and *T. harzianum* altered the repellency of *M. anisopliae* against termites. These results enhance the understanding of the complex interactions among lower subterranean termites and various fungi living in their habitats (Figure 4).

Esenther et al. (1961) were the first reported that the brown-rotting fungus, *Gloeophyllum trabeum* (Pers.) attracted subterranean termites *R. flavipes*, *Reticulitermes virginicus* (Banks), and *Nasutitermes columbicus* (Holmgren). After that, various wood-rotting fungi including *Phanerochaete chrysosporium* (Burdsall) and *Marasmiellus troyanus* (Murrill) (Amburgey, 1979; Ohmura et al., 1995; Cornelius et al., 2002, 2004, 2012), as well as blue-staining fungi including *Ophiostoma ips* (Rumb) and *Leptographium terebrantis* (Barras) (Little et al., 2012a, b, 2013; Clay et al., 2017), were reported to enhance aggregation and feeding activities of subterranean termites. These fungi may provide the chemical cues for termites to locate and make tunnels toward food sources. For example, the chemical (*Z*,*Z*,*E*)-3,6,8-dodecatrien-1-ol produced by *G. trabeum* has been identified as the major component of trail-following pheromone in various termite species such as *Reticulitermes lucifugus grassei* Clément, *Reticulitermes santonensis* Feytaud, and *Reticulitermes hesperus* Banks (Matsumura et al., 1976; Laduguie et al., 1994; Wobst et al., 1999; Saran et al., 2007). In addition, the presence of wood-rotting fungi could bring nutritional benefits to termites by providing proteins and improving their ability to metabolize lignocellulose (Waller and La Fage, 1987; Brune and Dietrich, 2015; Peterson and Scharf, 2016). On the contrary, termites may negatively affect wood-rotting fungi. Martin and Bulmer (2018) reported that subterranean termites can produce β-1,3-glucanases and have antifungal properties which suppress the growth of *G. trabeum* and *P. chrysosporium* (Burdsall).

In the present study, we found that almost all tested *Trichoderma* species (except *T. koningii*) triggered termite aggregation. These results indicate that fungi in the genus *Trichoderma* may exert generally attractive effects on *C. formosanus.* This attractive effect might be concentration-dependent, because *T. atroviride* with the concentration of 2.5 × 10^7^ conidia/g soil significantly attracted termites (Table 3), whereas the same fungus with the concentration of 1 × 10^7^ conidia/g soil showed no significant attractive effect (Table 6). However, the attractiveness of *T. longibrachiatum* and *T. harzianum* were consistent at both concentrations. Interestingly, *T. harzianum*, *T. hamatum*, *T. atroviride*, *T. virid*e, and *T. spirale* were attractive in the aggregation-choice tests but not active in the tunneling-choice tests. After releasing into open-air areas of the aggregation-choice tests, volatile chemicals produced by these *Trichoderma* species may elicit the olfactory responses of termites, and cause the aggregation preference. During tunnel excavating, however, termites contact the substrate with their cuticles and carry the sand particles with mouthparts. During these processes, haptic and/or gustatory cues may be needed to trigger tunneling preferences. Su (2005) reported that *C. formosanus* and *R. flavipes* made tunnels toward the wood discs infested with *G. trabeum*. To be detected by termites, the attracting chemicals produced by *G. trabeum* should be water-soluble and can “permeate through wet sand” (Su, 2005). Likewise, our study showed that termites produced significantly larger areas of tunnels in sand treated with *T. longibrachiatum* or *T. koningii*. These fungi may produce water-soluble chemicals, which provide haptic and/or gustatory cues to enhance the tunneling activities of termites. It would be valuable to investigate the underlying mechanisms of the aggregation and tunneling preferences triggered by different *Trichoderma* species.

Biedermann and Vega (2020) reviewed the evolution of insect-fungus mutualisms, which were driven by exchanged services such as nutrition and protection. The aggregation and tunneling preferences might be a result of positive selection because *Trichoderma* species benefit termites in many aspects. Mankowski et al. (1998) reported that consuming wood infested with *T. viride* increased the number of protozoa in the gut of *Zootermopsis angusticollis* (Hagen). Jayasimha and Henderson (2007) reported that *Trichoderma* species isolated from the cuticles and/or guts of *C. formosanus* suppressed the growth of *G. trabeum*, and therefore may help termites to compete for cellulose. Our study also showed that *Trichoderma* species play a role in the disease-defending processes of termites, because the three tested *Trichoderma* species not only suppressed the growth of *M. anisopliae* in the antagonism tests, but also significantly delayed the lethal effects of *M. anisopliae* in the mortality tests. Our study provides a novel example of environmental fungi associated with termites that reduce epizootic events. Likewise, some recent studies showed that *Streptomyces* spp. isolated from the gut and nest material of termites inhibited *M. anisopliae* under both *in vivo* and *in vitro* conditions (Chouvenc et al., 2013; Arango et al., 2016). Chouvenc et al. (2011) reviewed researches on biological control of termites in the past 50 years. Although many studies showed the potential of biological control as “environmentally friendly methods to control termites,” there is “little evidence to support practical applications in the field” (Chouvenc et al., 2011). The presence of *Trichoderma* spp. and *Streptomyces* spp. in the habitats of termites, which antagonize entomopathogenic fungi such as *M. anisopliae*, may partially explain the failure of termite biological control under field conditions.

In our mortality tests, the three tested *Trichoderma* species did not completely protect termites from the lethal effect of *M. anisopliae*, because the mortality of termites exposed to both conidia of *Trichoderma* species and *M. anisopliae* was not significantly different from *M. anisopliae* alone after 16 days. It is important to note that here a relatively high concentration of *M. anisopliae* was introduced. *M. anisopliae* produces various enzymes (e.g., proteases, chitinases, and lipases) and toxins (e.g., destruxins) that cause the death of insect hosts (Hu et al., 2006; Schrank and Vainstein, 2010). Although *Trichoderma* species can inhibit *M. anisopliae*, they may not be able to inactivate the enzymes and toxins produced by *M. anisopliae*, which may eventually cause the death of termites. In the field soils, the concentrations of *M. anisopliae* conidia are variable. Rath et al. (1992) isolated *M. anisopliae* strains from 419 samples of pasture soil in Tasmania, and found that the density for each strain was ranging from 1 × 10^2^ to 5 × 10^5^ colony forming units (cfu) g^–1^ soil. Nishi and Sato (2019) reported that the density of *Metarhizium* spp. in the rhizospheres of wild plants can reach to 4.2 × 10^6^ cfu g^–1^ dried soil. Weiser and Matha (1986) reported that the local densities of *M. anisopliae* can be very high because one corpse of infected insects can produce and release ∼10^10^ conidia in soil. One potential limitation of this study is that we only tested one conidial concentration of *M. anisopliae* and/or *Trichoderma* species. Since the field densities of these fungi may vary with habitats and environmental conditions, it is important to test the protective effect of *Trichoderma* species on termites at different conidial concentrations and *Trichoderma*/*Metarhizium* ratios in future studies.

Termites also have evolved many other defense mechanisms to reduce the risk of epizootic events (Myles, 2002; Liu et al., 2015, 2019a,b; Peterson and Scharf, 2016; Davis et al., 2018). Previous studies have shown that termites can produce various anti-microbial agents including termicin and spinigerin (Lamberty et al., 2001; Bulmer et al., 2009; Hamilton et al., 2011). In addition, behavioral immunity such as mutual grooming can effectively remove conidia of entomopathogenic fungi from cuticles and therefore protect termites (Shimizu, 2003; Yanagawa et al., 2005; Yanagawa and Shimizu, 2007). Many termites can also avoid to aggregate or make tunnels in the substrate containing harmful fungi (Staples and Milner, 2000; Mburu et al., 2009, 2011; Hussain et al., 2010; Sun et al., 2014). This “spatial avoidance” was also observed in our study, because *M. anisopliae*-treated sand significantly decreased aggregation and tunneling activities of termites. Interestingly, the presence of *T. longibrachiatum* or *T. harzianum* altered the repellent effects of *M. anisopliae* against termites in the aggregation-choice tests. The results of this study suggest that these *Trichoderma* species may increase the contact between termites and pathogens, and therefore negatively affect the health and survival of termites. The volatiles associated with these *Trichoderma* species likely disturb the olfactory responses of termite reacting to the *M. anisopliae* conidia. Screening such volatiles would help to break the spatial avoidance of entomopathogenic fungi by termites.

## Conclusion

Previous studies have shown complex interactions among termites and environmental microbes (i.e., wood-rotting fungi, blue-staining fungi, and entomopathogenic fungi). In the present study, nearly all *Trichoderma* species tested significantly increased aggregation of *C. formosanus*. *Trichoderma* species had antagonistic effects against *M. anisopliae*, and protected termites from infection. Although *M. anisopliae* conidia repelled termites, the presence of *T. longibrachiatum* or *T. harzianum* reduced the repellency of *M. anisopliae* against termites.

## Data Availability Statement

The raw data supporting the conclusions of this manuscript will be made available by the authors, without undue reservation, to any qualified researcher.

## Ethics Statement

Ethical review and approval was not required for the animal study because the animals used in this study were termites, a very destructive pest. Distress to the termites during their collection in the field and their transport to the laboratory was minimized as much as possible. In the laboratory, colonies were maintained under suitable conditions, thereby maximizing their welfare and survival. After the end of the experiments, colonies were kept in the laboratory until their natural death.

## Author Contributions

CaW and ChW conceived and designed the experiments. ChW and HX performed the experiments. ChW was mainly responsible for analyzing the data and writing the manuscript. CaW, XW, and JW were involved in the revision of the manuscript.

## Conflict of Interest

The authors declare that the research was conducted in the absence of any commercial or financial relationships that could be construed as a potential conflict of interest.

## Footnotes

1 http://www.ncbi.nlm.nih.gov/BLAST/
